# Grief and Avoidant Death Attitudes Combine to Predict the Fading Affect Bias

**DOI:** 10.3390/ijerph15081736

**Published:** 2018-08-13

**Authors:** Jeffrey A. Gibbons, Sherman A. Lee, Ashley M.A. Fehr, Kalli J. Wilson, Timothy R. Marshall

**Affiliations:** 1 Department of Psychology, Christopher Newport University, 1 Avenue of the Arts, Newport News, VA 23606, USA; sherman.lee@cnu.edu (S.A.L.); kalli.wilson.11@cnu.edu (K.J.W.); tmarshal@cnu.edu (T.R.M.); 2 Department of Psychology, Old Dominion University, 5115 Hampton Blvd, Norfolk, VA 23529, USA; amafehr@gmail.com

**Keywords:** fading affect bias, complicated grief, death attitudes

## Abstract

The fading affect bias (FAB) occurs when unpleasant affect fades faster than pleasant affect. To detect mechanisms that influence the FAB in the context of death, we measured neuroticism, depression, anxiety, negative religious coping, death attitudes, and complicated grief as potential predictors of FAB for unpleasant/death and pleasant events at 2 points in time. The FAB was robust across older and newer events, which supported the mobilization-minimization hypothesis. Unexpectedly, complicated grief positively predicted FAB, and death avoidant attitudes moderated this relation, such that the Initial Event Affect by Grief interaction was only significant at the highest 3 quintiles of death avoidant attitudes. These results were likely due to moderate grief ratings, which were, along with avoidant death attitudes, related to healthy outcomes in past research. These results implicate complicated grief and death avoidant attitudes as resiliency mechanisms that are mobilized during bereavement to minimize its unpleasant effects.

## 1. Introduction

People undergo a host of psychological challenges in the wake of a significant loss, even though most individuals recover quickly [1]. One indicator of healthy bereavement may involve the phenomenon referred to as the Fading Affect Bias (FAB) [2,3,4], where unpleasant emotions fade faster than pleasant emotions [5,6,7]. The literature on the FAB has examined the phenomenon across a variety of events, including significant/death and non-significant/everyday events [8]. However, studies have not examined the relation of bereavement outcomes to the FAB for these types of events. Such an investigation may reveal mechanisms involved in bereavement. Therefore, we designed the current study to examine the relation of FAB to three main bereavement related outcomes: complicated grief, negative religious coping, and death attitudes.

### 1.1. Fading Affect Bias

Research has shown that participants recorded and recalled pleasant events more often than unpleasant events [9,10,11,12]. Unpleasant events also lost their emotional intensity faster than pleasant events [13,14]. Walker et al., (1997) found similar differential fading affect that increased with retention interval in diary studies for college students who recalled events after 3 months, 1 year, or 5 years [7]. Walker et al., (2003a) later deemed this pattern of fading affect the fading affect bias (FAB) [3]. Recent research showed consistent FAB effects across event types [15,16], which suggests that the FAB is a general, healthy/pleasant coping outcome. As the FAB is a healthy outcome, some researchers argue that it may enhance pleasantness [4,17] by helping people pursue pleasant experiences and avoid unpleasant ones [2].

The research on the FAB supports theories emphasizing positive psychology, because the phenomenon increases with pleasant/healthy outcomes and decreases with unpleasant/unhealthy outcomes. One such positive psychology theory is the mobilization-minimization hypothesis [18], in which mental and physical resources are galvanized and employed to diminish the effects of negative events and emotions. In support of this theory, the FAB is positively related to positive mechanisms, such as social rehearsals [19,20,21]. Conversely, the FAB is disrupted by unpleasant emotions, such as dysphoria [3], dispositional mood [22], and trait anxiety [23]. Although FAB research has examined the phenomenon in the context of death [8,24,25], it has not examined the relation of the FAB to grief for death events.

### 1.2. Complicated Grief, Negative Religious Coping, and Death Attitudes

Death of a spouse and death of a close family member rank among the top five most stressful events in life, alongside divorce, separation, and imprisonment [14]; the effects of death are particularly strong for family members who report a strong need of social support to overcome or avoid outcomes, such as poor work performance [26,27] and strong emotional reactions, such as grief [28]. Difficulties in the bereavement process are associated with three notable factors: complicated grief, negative religious coping, and death attitudes.

Complicated grief describes a maladaptive pattern of adjustment to loss that is distinct from other forms of psychopathology, such as depression, anxiety, and post-traumatic stress disorder [29]. Symptoms of complicated grief include such experiences as bitterness over the loss, pre-occupation with thoughts of the deceased, and yearning for the deceased [30]. These symptoms involve a wide range of adverse mental, physical, and social outcomes [29,31]. Because cognitive impairments, such as rumination [32], global cognitive deficits [33], and reduced autobiographical memory specificity [34], seem to be tied to complicated grief, we predicted a similar disruption for FAB in the current study.

Complicated grief symptoms can be a maladaptive part of grieving, which may be enhanced by negative religious coping. Negative religious coping describes a maladaptive process that involves feelings of confusion about one’s faith, abandonment by God, and reinterpretations of outcomes as acts of the Devil’s or God’s punishment [35]. This spiritual struggle is connected to a range of life problems [36] as well as dysfunctional grief symptoms [37,38,39]. As negative religious coping reflects difficulties in adjustment to loss and disruptions in the FAB for death events [18], we hypothesized that it would negatively predict FAB in the current study.

Death attitudes also predict bereavement and they may predict FAB as well. Attitudes about death influence the perceptions of death events [40,41], they help guide emotions [42], and they include death avoidance and death acceptance [43]. Death avoidance involves refusal to accept the certainty of death and it can lead to negative emotions, such as anxiety [40] and grief [44]. Shear et al., (2007) determined that avoidant behavior in response to grief predicted poor perceived health and psychological distress [45]. In sum, death avoidance attitudes contribute to the bereavement process. Conversely, people who adopt death acceptance attitudes embrace the reality and unavoidability of death [46], and they show high optimism, well-being [40,43], and life satisfaction [47]. Based on these findings, we expected a negative relation for death avoidance and FAB and a positive relation for death acceptance and FAB.

## 2. The Current Study

**Figure 1:**
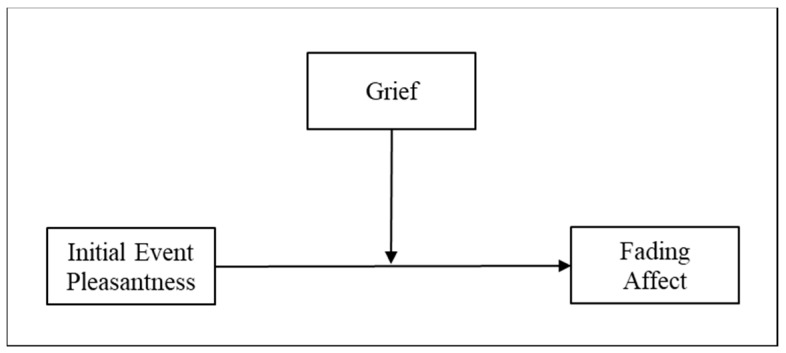
Two-way interaction between grief and initial event affect in predicting fading affect.

A growing literature suggests that the FAB is a healthy coping outcome that may help people seek pleasant experiences [2] in several contexts, including death [8]. The research on FAB research supports theories stressing positive outcomes and resiliency mechanisms, such as the mobilization-minimization hypothesis [18], where mental resources are used to reduce the impact of negative events and emotions. Based on the importance of complicated grief symptoms, negative religious coping, and death attitudes for bereavement in the thanatology literature, we investigated their ability to disrupt the FAB in the context of death. Because previous research has shown that neuroticism [48], depression, anxiety, and stress [31,32] also influence bereavement, we included these variables in our analyses as well. Specifically, we examined FAB in the context of death and evaluated time and grief as its main moderators while controlling for the remainder of the individual difference variables (Figure 1).

**Figure 2:**
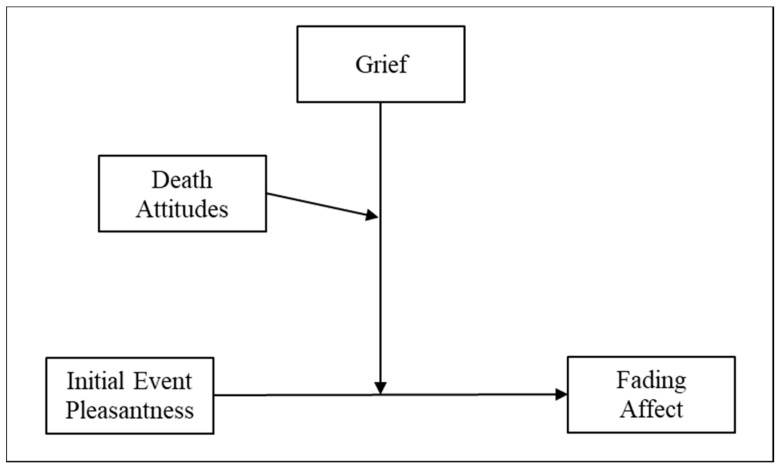
Three-way interaction between grief, initial event affect, and avoidant death attitudes in predicting fading affect.

We expected strong/robust FAB effects and we expected FAB effects to be larger with older events than with newer events. We also expected grief to negatively predict FAB. In the interest of thoroughness, we examined each of the remaining individual difference variables as moderators of FAB while controlling for the other ones. We expected each of those variables, except mature/accepting death attitudes, to negatively predict FAB. We also examined the potential for each of the individual difference variables to moderate (1) the relation of FAB and time, as well as (2) the relation of FAB and grief, while controlling for the other variables (Figure 2). We expected complicated grief, negative religious coping, and death attitudes to play the strongest moderating roles for FAB.

## 3. Method

### 3.1. Participants

The current study included responses from 198 undergraduate students at a small southeastern liberal arts university. Most of the participants were White (80.80%), women (76.00%), and ranged in age from 18 to 30 years old (*M* = 19.63, *SE* = 0.10). We recruited participants from introductory psychology courses and gave them course credit for their participation, as applicable. Participants followed the guidelines of the American Psychological Association (APA) [49]. Specifically, participants were briefed about the procedures that they would follow in the experiment before it began, and they were asked to provide informed consent. Participants were informed that they could leave the study at any time and they were given the number for the university counseling center in case they experienced troublesome emotions. After the experimental procedures had been completed, participants were debriefed about the general goals of the study.

### 3.2. Materials and Measures

Many of the measures used in the Gibbons et al., (2016) study were also used in the current study [8]. We described the important predictors, control variables, and the outcome variable, fading affect, in the following subsections.

**Grief.** The Inventory of Complicated Grief assesses symptoms of complicated grief [30] using 19 items. Items are rated from 1 (never) to 5 (always) with high scores indicating strong symptoms of complicated grief. Examples of items include “I felt myself longing for the person who died” and “I felt bitter over this person’s death”. Cronbach’s alpha for the grief scale was 0.94.

**Religious coping.** To assess religious coping for unpleasant events, we used the 14-item Brief RCOPE from Pargament et al., (1998) [36]. Questions were rated on a Likert-type scale ranging from 1 (not at all) to 4 (a great deal). We administered the questions in the past tense considering that students retrieved all events from memory. Both positive and negative subscales were included with items, such as “Sought God’s love and care” for positive coping and “Questioned God’s love for me” for negative coping. As indicated, positive religious coping implies a positive and secure relationship with God or one’s deity, whereas negative religious coping is an ominous and tenuous view of the world and of one’s relationship with God. We calculated average scores for positive and negative religious coping across everyday events and significant events. Cronbach’s alpha for positive and negative religious coping were 0.96 and 0.91, respectively.

**Death acceptance and death avoidance attitudes.** Klug and Sinha’s (1987–1988) 16-item Death Acceptance Scale (DAS) measured the meaning that a participant finds in death as well as his/her death attitudes in the current study [46]. This scale assesses both confrontation and integration of death experiences in daily life. For each component, eight items were included. Questions indicating acceptance included such items as “I enjoy life more as a result of facing the fact of death”. We assessed avoidance with items, such as “I really prefer not to think about death”. Items were rated on a Likert-type scale, ranging from 1 (strongly agree) to 4 (strongly disagree). A two-factor, principal components factor analysis and a reliability analysis showed that death acceptance included seven items (items 2, 3, 8, 9, 11, 12, and 15) and death avoidance included eight items (items 1, 4, 5, 7, 10, 13, 14, and 16). We calculated average death acceptance and death avoidance scores with high scores indicating high acceptance and high avoidance, respectively. Cronbach’s alpha for the death acceptance and death avoidance scales were 0.84 and 0.76, respectively.

**Neuroticism.** The current study used the neuroticism/emotional stability subscale of the brief version of the Big Five, called Mini Markers [50]. The questionnaire asks participants to rate the extent that they believe in the accuracy of self-descriptive adjectives (e.g., envious), on a 9-point Likert-type scale, ranging from 1 (extremely inaccurate) to 9 (extremely accurate). We reverse scored items when necessary, and then we calculated their average score. High scores indicated high neuroticism. Cronbach’s alpha for the neuroticism scale was 0.79.

**Depression, anxiety, and stress.** The Depression, Anxiety, Stress Scale (DASS-21) assesses three negative emotional states [51], with three seven-item questionnaires for a total of 21 items pertaining to depression, anxiety, and stress. All items are scored on a 4-point scale, ranging from 0 (did not apply to me at all) to 3 (applied to me very much or most of the time). We calculated average scores for depression, anxiety, and stress with high scores indicating strong psychological stress. An example of a depression item is “I found it difficult to work up the initiative to do things”. An example of an anxiety item is “I felt I was close to panic”. An example of a stress item is “I found it hard to wind down”. Cronbach’s alphas for depression, anxiety, and stress were 0.86, 0.82, and 0.80, respectively.

**Closeness.** Previous research has shown that closeness to deceased is related to bereavement [52]. Therefore, an individual’s closeness to the deceased was assessed using the Inclusion of Other in Self (IOS) Scale [53]. This scale includes one pictorial item with seven possible choices. A pair of circles begin far apart and move closer together across the seven choices, indicating high closeness, and the participant selects the option that depicts an accurate closeness of the relationship in question. Although the scale only uses one item, the IOS demonstrated high test-retest reliability and convergent validity with the Relationship Closeness Inventory [54] and other measures of closeness in past studies.

**Figure 3:**
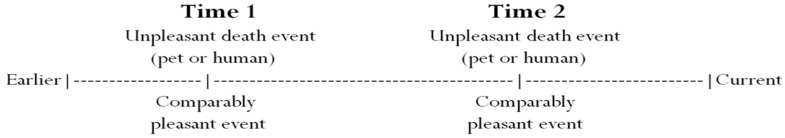
Events timeline for the unpleasant death events of a pet or human and matching comparably pleasant events both at Time 1 and Time 2.

**Fading affect.** The questionnaire prompted participants to describe four events that occurred within 5 years of the test date. The four events included two death events: a recent death event and a death event that occurred before that event. Participants could describe the death of family, a friend, an acquaintance, or a pet. We asked participants to describe two pleasant events that occurred around the time of the two death events and that showed comparable initial affective intensity as the death events (see Figure 3 for the events timeline). For the significant pleasant events, many people reported events, such as the birth of loved ones, graduations, or weddings. For each event, participants reported the month and year in which the event occurred and wrote a short description of the event, disclosing only as much information as they felt comfortable sharing. Participants reported the way they felt at the time the event occurred (i.e., their original emotion) and the way they felt at the time of testing (i.e., their current emotion) using two pleasantness scales ranging from −3 (very unpleasant) to +3 (very pleasant), including a score of 0 (neutral). For initially pleasant events, we calculated fading affect by subtracting the current affect from the original affect. For initially unpleasant events, we calculated fading affect by subtracting the original affect from the current affect. These calculations insured that all measures of fading affect were positive across pleasant and unpleasant events, such that a large fading affect score indicated a large amount of fading, and a small fading affect score indicated very little fading. As in most FAB studies, fading affect was the main dependent variable or criterion in the current study. We used the other variables in the study to predict fading affect.

### 3.3. Procedure

After signing up for the study, participants received a webpage link to complete Phase 1 of the study online. That is, participants completed half of the questionnaires on Google Forms, including demographics and the scales for Big 5 and current Death Attitudes. Participants were required to create a codename for the researchers to match Phase 1 (online) and Phase 2 (in-person) responses. Next, participants came in-person for Phase 2 to complete remaining questionnaires, including the DASS-21 and responses to four events. We explained the difficult or confusing portions of the instructions in detail to participants using a written script. We then collected information from participants regarding a series of four specific life events occurring in the last 10 years.

Participants were asked to report events earlier in time (Time 1) and then later in time (Time 2). Events for both times each included (1) an unpleasant death event of a pet or someone close and (2) a pleasant event comparable to the death event in emotional intensity. In addition, participants had to report on their emotion concerning the Time 1 death event at the time the Time 2 death event occurred (see Figure 3 for the events timeline). This repeated-measures instructional manipulation occurred within a cross-sectional retrospective study. We instructed participants to provide significant pleasant events that were comparable in initial emotional intensity to the death event. Specifically, if a participant rated his/her death event as a −3 on the scale, a comparable pleasant significant event needed a rating of +3. For the significant pleasant events, many people reported events such as the birth of loved ones, graduations, or weddings.

For each event, participants reported the month and year in which the event occurred and wrote a short description of the event, disclosing only as much information as they felt comfortable sharing. Participants then reported the way they felt at the time the event occurred (i.e., their original emotion) and the way they felt at the time of testing (i.e., their current emotion) using two pleasantness scales. For death events, participants completed the 14-item brief religious coping scale to assess the frequency that certain religious behaviors were used to cope with the specific unpleasant life events. Participants also completed responses to scales on closeness, complicated grief, general coping, and death attitudes for the event that they were describing (either Time 1 or Time 2). Participants completed all questionnaires in approximately 90 min and then they were debriefed and informed about free counseling provided by the university.

### 3.4. Analytic Strategy

We first used parametric statistics to examine fading affect across initial event affect and time, while controlling for a nominal-level variable to represent each participant as well as several individual difference variables (e.g., grief, neuroticism, depression, anxiety, stress, negative religious coping, avoidant death attitudes, mature/accepting death attitudes). We then used non-parametric statistics to examine moderators for the FAB. Specifically, we tested each of the two-way interactions of initial event affect (pleasant vs. unpleasant) and an individual difference variable (e.g., grief) in predicting fading affect, while controlling for relevant main effects, participant, time (older and newer event), and the other individual difference variables. We also tested the three-way interactions between initial event affect, event time, and an individual difference variable (e.g., grief), while controlling for all two-way interactions, relevant main effects, the participant, as well as the other individual difference variables. As grief was the main predictor variable for fading affect after initial event affect, we also tested the three-way interactions between initial event affect, grief, and each of the remaining continuous individual difference variables, while controlling for the participant and time variables, as well as the other individual difference variables.

We employed Model 1 of the Process macro via IBM SPSS [55] to examine fading affect, *y*, among pleasant and unpleasant events, *x*, across the continuum of each individual difference variable (e.g., grief) as the moderator, *m*, of the FAB (relation of *x* and *y*), while controlling for participant, time variables, and the other individual difference variables. We then used Model 3 (Hayes 2013) to evaluate the effect of initial event affect, *x*, on fading affect, *y*, among older and newer events, *w*, conditional upon levels of the self-reported individual difference variables, *m*, such as grief, while controlling for the participant and time variables, as well as the other individual difference variables. We also used Model 3 to evaluate the effect of initial event affect, *x*, on fading affect, *y*, across the continuum of grief, *w*, conditional upon levels of each of the other individual difference variables, *m*, while controlling for participant and time, as well as the remaining individual difference variables. When examining the three-way interactions, we employed the Johnson-Neyman technique in the Process macro to examine the interactive effect of initial event affect, *x*, and either time or grief, *w*, on fading affect, *y*, across levels of the other individual difference variables, *m*, as moderators [36]. This technique determines the exact point along the continuum of those moderator variables where the FAB becomes significant, rather than drawing an arbitrary line to determine “low” and “high” groups [56].

## 4. Results

### 4.1. Correlations Between Predictors

Fading affect did not correlate to the other measures in the study because we calculated it for pleasant and unpleasant events. Therefore, Table 1 presents the correlations between the individual difference variables. Except for mature/accepting death attitudes, the individual difference variables positively correlated with magnitudes ranging from small to moderate.

### 4.2. Justifying the Grouping of All Death (Initially Unpleasant) Events

Participants provided many more human death events (*n* = 210) than pet death events (*n* = 62) and unlabeled death events (*n* = 20). Therefore, we wanted to combine the events into a single category of death events. We examined closeness for each event and the absolute value for the initial pleasantness for events to provide evidence that the pet deaths were just as important and initially emotionally intense as human deaths. Participants were just as close to pets that died as they were to humans who died because the participants rated the pets in pet deaths as closer than the individuals in other deaths. Moreover, the absolute value of the initial pleasantness for all four events (three types of death events and regular events) was not different. These results suggested that the deaths were not fundamentally different, and they could be combined into a single category of death events without bias.

**Comparing closeness across death events**. Levene’s test of equality of variance was statistically significant, *F*(2, 284) = 4.27, *p* = 0.015, but heterogeneity of variance is not a serious problem until the *F*-value exceeds 9.00 [57]. The overall analysis of variance for closeness was statistically significant, *F*(2, 276) = 3.57, *p* < 0.001, *η*^2^*_partial_* = 0.12, and the main effect of death type was statistically significant, *F*(2, 276) = 11.30, *p* < 0.001, *η*^2^*_partial_* = 0.08. A set of LSD *t*-tests showed that the pets that died were closer to participants (*M* = 5.55, *SE* = 0.22) than the humans who died (*M* = 4.37, *SE* = 0.12) and the individuals in deaths that were not labeled (*M* = 4.19, *SE* = 0.42). However, the same tests revealed no difference in closeness for human deaths and unlabeled deaths.

**Comparing initial pleasantness across all events (missing data excluded)**. The participants provided initial affect ratings that matched the instructions for 582 events (73.48%). The overall analysis of variance examining the absolute value of initial pleasantness was not statistically significant, *F*(11, 570) = 0.86, *p* = 0.58, *η*^2^*_partial_* < 0.02, and the main effect of event type was not statistically significant, *F*(3, 570) = 1.44, *p* = 0.23, *η*^2^*_partial_* = 0.008. Specifically, initially pleasant events (*M* = 2.53, *SE* = 0.04), human deaths (*M* = 2.41, *SE* = 0.05), pet deaths (*M* = 2.42, *SE* = 0.09), and unlabeled death events (*M* = 2.35, *SE* = 0.17) were not rated differently on their initial pleasantness.

### 4.3. Missing Data for Initial Pleasantness and Fading Affect

The focus of the current study was to examine fading affect for the 792 events provided by 198 participants. Most of the events changed in affect or showed no change in affect, but some events increased in affective intensity (*n* = 34, 4.29%), which is referred to as flourishing affect [17]. Other events switched their affective intensity to affect that was the opposite of the initial event affect (*n* = 78, 9.85%), which is referred to as changed affect [22]. The flourishing and changed affect events overlapped with three of the five mislabeled/unrated events and the data for these events were removed from the analyses (*n* = 114, 14.39%), because these affective changes should not be included in calculations of affect that is supposed to fade [8]. Importantly, participants either did not provide affect ratings for some events or they provided the wrong initial affect rating for events (*n* = 226, 28.50%). This large portion of missing data indicated the need to examine whether this loss occurred randomly or systematically.

Missing data are only a concern when they are missing systematically [58,59,60]. To test the degree that missing data were missing at random in the current study, Little’s Missing Completely at Random (MCAR) test was run on the following variables: fading affect, absolute value of initial pleasantness, depression, anxiety, stress, neuroticism, grief, avoidant death attitudes, and accepting death attitudes. For the combination of these variables, Little’s MCAR test was significant (χ^2^ (32) = 151.390, *p* < 0.0001), indicating that the missing data were not missing completely at random. Dong and Peng (2013) noted that such data are notoriously biased and inefficient [61]. Therefore, we used the expectation-maximization (EM) method [62] to replace the missing values [63].

### 4.4. Initial Pleasantness Across Event Affect and Event Time for All Viable Events

The overall analysis of variance was statistically significant, *F*(11, 666) = 1.99, *p* = 0.027. With the exception of negative religious coping, *F*(1, 666) = 7.75, *p* = 0.006, *η*^2^*_partial_* = 0.012, the effects for these control variables were not significant. The main effect of initial event affect was statistically significant, *F*(1, 666) = 6.92, *p* = 0.009, *η*^2^*_partial_* = 0.010, such that initially pleasant events (*M* = 2.63, *SE* = 0.03) were rated as more initially intense than initially unpleasant events (*M* = 2.52, *SE* = 0.03). This result indicates that any FAB effects cannot be the result of regression to the mean. The main effect of event time was not statistically significant, *F*(1, 666) = 0.21, *p* = 0.64, *η*^2^*_partial_* < 0.001, and the Initial Event Affect x Event Time interaction was not statistically significant, *F*(1, 666) = 0.82, *p* = 0.365, *η*^2^*_partial_* = 0.001.

### 4.5. Fading Affect and the Influence of Initial Event Affect and Event Time

**Figure 4:**
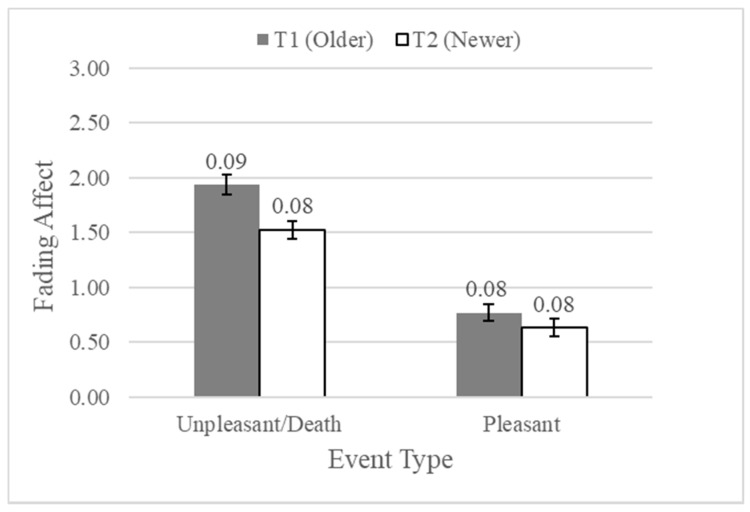
Fading affect for both unpleasant (death) and pleasant events at Time 1 (older) and at Time 2 (newer).

The overall analysis of variance was statistically significant, *F*(11, 666) = 1.99, *p* = 0.027. Of the control variables, the effects for negative religious coping, *F*(1, 666) = 5.96, *p* = 0.015, *η*^2^*_partial_* = 0.009, and avoidant death attitudes, *F*(1, 666) = 11.32, *p* = 0.001, *η*^2^*_partial_* = 0.017, were significant. Moreover, the main effect of initial event affect was statistically significant, *F*(1, 666) = 139.14, *p* < 0.001, *η*^2^*_partial_* = 0.173, such that the affect for initially unpleasant events (*M* = 1.51, *SE* = 0.04) faded more than the affect for initially pleasant events (*M* = 0.83, *SE* = 0.04), which demonstrates the expected FAB. In addition, affect faded more for older events (*M* = 1.26, *SE* = 0.04) than for newer events (*M* = 1.09, *SE* = 0.04), *F*(1, 666) = 8.70, *p* = 0.003, *η*^2^*_partial_* = 0.013, which supported the findings in the literature. The two-way interaction between initial event affect and event type was not statistically significant, *F*(1, 666) = 1.76, *p* = 0.186, *η*^2^*_partial_* = 0.003 (Figure 4), but affect faded significantly more for older unpleasant events than for newer unpleasant events, even though affect did not fade differently for older pleasant events than for newer pleasant events. These results partially support the hypothesis about FAB and time.

### 4.6. Fading Affect and the Influence of Grief

**Figure 5:**
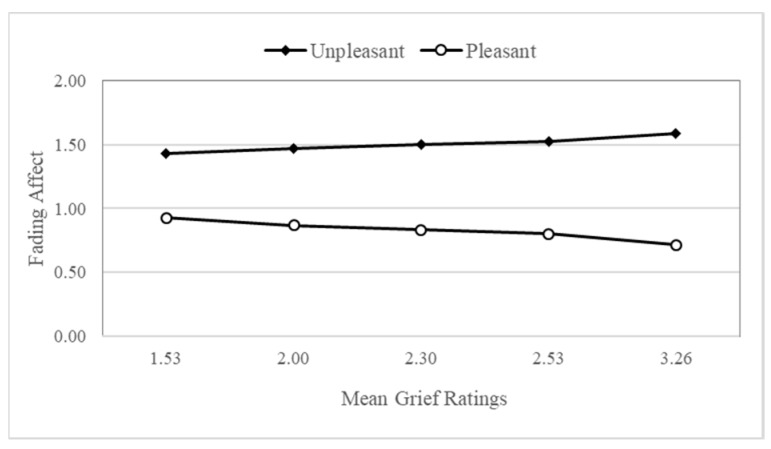
Fading affect for initial event affect across quintile levels (10th through 90th) of grief ratings for events.

In contrast to the previous results, we used non-parametric statistics to analyze fading affect. The results from the Process Model 1 [55] revealed main effects for time, initial event affect, grief, negative religious coping, avoidant death attitudes, and accepting death attitudes. Moreover, Model 1 showed one significant two-way interaction between initial event affect and grief, B = 0.22 (*SE* = 0.09), *t* (665) = 2.34, *p* < 0.035, 95% CI (0.04, 0.40), Δ*R*^2^ = 0.007, such that the FAB increased along with grief. Figure 5 depicts this interaction, showing the fading affect for pleasant events and the fading affect for unpleasant events across each quintile of the grief measure scores. The direction of this relation was not expected. Table 2 includes the coefficients for the FAB across each of five quintiles for grief. As pleasant events were coded as 1 and unpleasant events were coded as 2, large, positive coefficients represent a strong FAB.

### 4.7. Three-Way Interaction: Initial Event Affect, Grief, and Avoidant Death Attitudes

**Figure 6:**
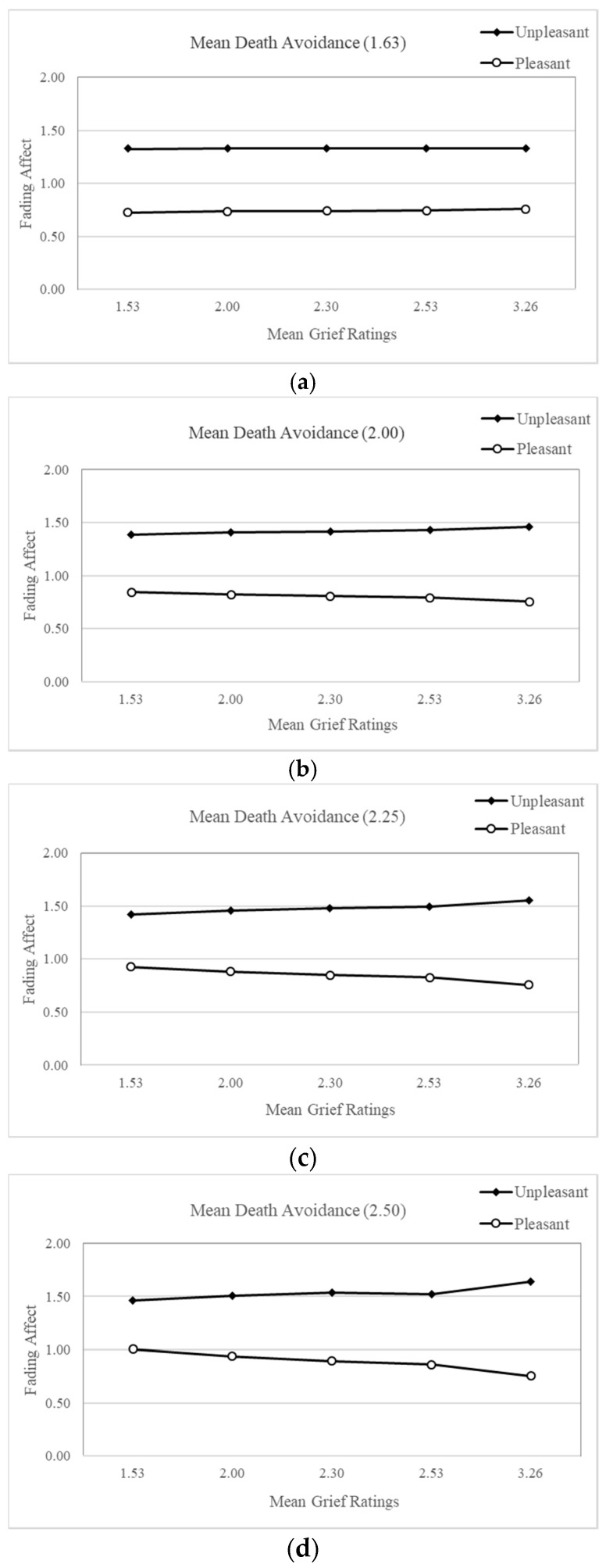

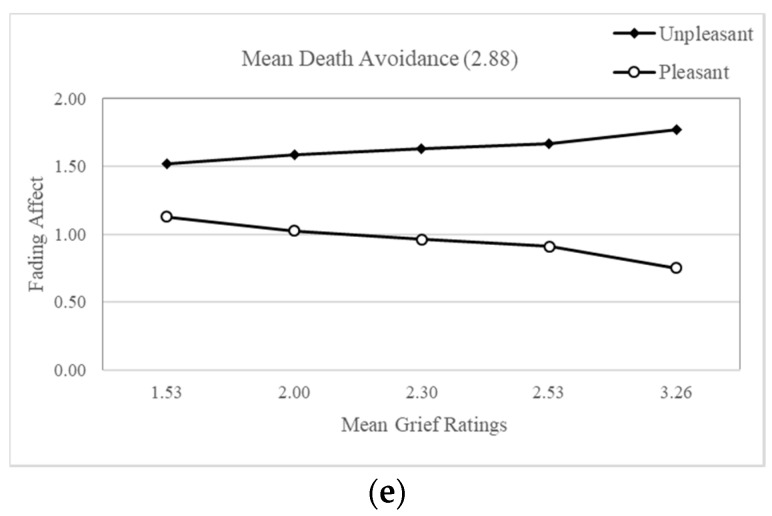
(**a**) Fading affect for initial event affect across quintile levels (10th through 90th) of grief ratings for events at mean avoidant death attitudes of 1.630 for events; (**b**) Fading affect for initial event affect across quintile levels (10th through 90th) of grief ratings for events at mean avoidant death attitudes of 2.000 for events; (**c**) Fading affect for initial event affect across quintile levels (10th through 90th) of grief ratings for events at mean avoidant death attitudes of 2.252 for events; (**d**) Fading affect for initial event affect across quintile levels (10th through 90th) of grief ratings for events at mean avoidant death attitudes of 2.500 for events; (**e**) Fading affect for initial event affect across quintile levels (10th through 90th) of grief ratings for events at mean avoidant death attitudes of 2.880 for events.

The results from the Process Model 3 [55] revealed only one significant three-way interaction, with death avoidant attitudes being the only continuous individual difference variable to moderate the interactive effect of initial event affect and grief to predict fading affect, B = 0.30 (*SE* = 0.14), *t*(662) = 2.15, *p* < 0.032, 95% CI [0.03, 0.58], Δ*R*^2^ < 0.006 (Figure 6a–e). Table 3 includes the coefficients for the FAB across the five quintiles of grief and the five quintiles of death avoidant attitudes, with large, positive coefficients representing large FAB. These results show that the FAB was not significant at low levels of grief and death avoidant attitudes, but it became significant at higher levels of both measures. Figure 6a shows that the FAB is not related to grief for the first quintile of death avoidant attitudes and Figure 6b shows that the FAB begins to be positively, albeit non-significantly, related to grief for the second quintile of death avoidant attitudes. Importantly, Figure 6c–e show that the FAB is significantly, positively, and progressively related to grief for the remaining quintiles of death avoidant attitudes. The Johnson-Neyman results indicated that the Initial Event Affect × Grief interaction became significant at the 0.05 level when death avoidant attitudes were 2.34 (Figure 6c), and the effect increased from there with death avoidant attitudes. The results for the three-way interaction support the hypothesis that grief and death attitudes would play the strongest moderating role for the FAB.

## 5. Discussion

Much like past research, we found a robust overall fading affect bias (FAB), such that unpleasant (death) events faded more than pleasant events [8], even though pleasant events were initially more intense. These results support the mobilization-minimization hypothesis, where individuals mobilize mental resources to reduce the effects of negative circumstances [18]. In addition, older death events showed greater fading affect and FAB than newer death events. This research replicated the work of Walker et al., (1997) who showed that the FAB, which is predominantly driven by the fading of unpleasant events, increased with time [7].

Moreover, we found an Initial Event Affect by Grief interaction, such that the FAB increased as grief increased. Specifically, death events, representing unpleasant events, faded more than pleasant events, and this differential fading affect increased as grief increased. This unexpected finding suggests that grief is positively related to healthy emotional outcomes in the context of death. Finally, we found that death avoidant attitudes moderated the Initial Event Affect by Grief interaction, such that the two-way interaction was not significant at low levels of death avoidant attitudes, but it became significant at medium levels of death avoidant attitudes and it increased at higher levels. The three-way interaction results suggest that grief positively relates to healthy emotional outcomes in the face of death, but such outcomes seem to depend on avoiding thoughts and emotions related to the circumstances surrounding these events. Together, the two- and three-way interactions indicate that grief and avoidant death attitudes may be resiliency mechanisms for bereaved individuals, which mobilize mental resources to minimize the effects of death [18].

The results in the current study could also be in line with the attention bias explanation [64] and the schema model [65]. Based on the attention bias explanation, most or many of the participants in the current study would have frequently experienced pleasant emotions and infrequently experienced unpleasant emotions in their past, which helped these individuals pay more attention to pleasant than unpleasant emotions. Consequently, participants in the current study maintained their pleasant emotions in memory and they let go of their unpleasant emotions. For the schema model, most or many of the participants in the current study would have had to experience close and positive emotional attachments with their parents, which created early adaptive schemas that maintained pleasant emotions and aided the degradation of unpleasant emotions. Future research could test these explanations by replicating the methods in the current study with two added features: use an emotion priming procedure to identify fast and slow processors of pleasant emotions and employ parental attachment questionnaires to identify proper or poor emotional parental attachments. The attention bias explanation gains credence for the results in the current study if the effects in the current study (e.g., FAB, positive relation of FAB and grief) are weak for slow processors of pleasant emotions, whereas the schema model is a viable account of the findings in the current study if its effects are weak for participants with poor emotional attachments to their parents and, hence, early and persistent maladaptive schemas.

At first glance, the positive relation between FAB and grief symptoms seems at odds with the proposal that the FAB represents a positive coping outcome [2] because grief is often associated with negative/unhealthy outcomes [28,66]. However, further inspection of the data provided an alternative perspective. First, we found moderate, positive associations between grief constructs and both negative religious coping and neuroticism (Table 1), replicating past grief research [37]. Second, the FAB effects in the current study replicated the effects found in previous FAB studies where unpleasant affect faded to a greater degree than pleasant affect and this effect was very robust [67]. These findings indicated that the positive relation between grief and the FAB reflects reality. We further analyzed the data in the current study and looked to the literature to make sense of this unexpected relation.

A descriptive analysis of the complicated grief symptom ranges in the current study revealed that the grievers were mostly composed of low to moderate level grievers (80% of ratings fell at or below the midpoint of the scale). Therefore, the “high” grief scores (upper 50%) in the current study represented moderate and not pathological levels of distress. This detail is important when evaluating the findings because past research has shown that moderate levels of grief are sometimes associated with positive outcomes. For example, Currier, Holland, and Neimeyer (2012) found that bereaved individuals who experienced intermediate levels of grief exhibited the greatest growth when compared to low and high grievers [68]. Grievers who report moderate grief may experience enough suffering to facilitate personal growth, which contrasts the minimal growth needed by low grieving grievers and the unlikely growth for high grieving grievers who are completely overwhelmed. Moreover, other researchers have found a positive association between grief symptoms and growth [69]. In fact, individuals who experience intense rumination and intrusions after a traumatic event tend to report strong levels of posttraumatic growth [70].

Like grief symptoms, negative cognitions are unpleasant, but they can also play a constructive role in helping individuals adjust from a stressful life experience. For instance, Helgeson, Reynolds, and Tomich (2006) found that intrusive and avoidant thoughts produced by a serious illness were positively related to benefit finding, which is a cognitive phenomenon linked to healthy outcomes [71]. Therefore, impairment does not always follow the negative cognitions produced during and after stressful life events. Conversely, early emotional processing during bereavement does not mean that delayed reactions can be avoided, as no evidence supports this ‘grief work’ proposal [72]. In fact, Bonnano and Field (2001) discovered in a longitudinal study of conjugally bereaved adults that low emotional processing of grief early in bereavement was not associated with a delayed future grief reaction [72].

As grief was positively associated with the FAB in the current study for individuals who generally reported no more than moderate grief ratings, factors, techniques and interventions that are positively related to the FAB may reduce grief for individuals who experience extremely low or extremely high grief. For example, mindfulness-based cognitive therapy reduces unpleasant affect and enhances pleasant affect and it may be able to reduce grief at the extremities [73]. Based on the finding that social rehearsals enhance the FAB [20], social interaction may reduce grief at the extremities because it retains positive affect, reduces relapses of depression [74], and it increases positive moods [75]. Of course, extreme grief could be addressed via conventional means, such as acceptance of painful feelings and memories related to bereavement, as it is important in understanding grief [76]. Future research should examine these possibilities, particularly for individuals who experience extremely low or extremely high grief.

The three-way interaction of initial event affect, grief, and avoidant death attitudes in predicting fading affect shows that the FAB does not appear for death events at low levels of grief and death avoidant attitudes but it is noticeable at higher levels of both measures. In other words, the Initial Event Affect by Grief interaction depends on avoidant death attitudes, such that the unexpected two-way interaction does not appear at low levels of death avoidant attitudes, but it is noticeable at medium and higher levels of the measure. This finding is in line with past research showing that avoidant thoughts are connected to healthy outcomes [71], and it suggests that avoidant thoughts facilitate cognitive functioning in the form of primed emotions [77,78] that help regulate emotions produced by death, which could enhance survival [2,79]. Therefore, every theoretical and real-world implication mentioned in the previous paragraphs for low to moderate grievers may depend on the degree that people do or do not avoid thoughts and feelings related to circumstances surrounding death events. The three-way interaction denotes the potential importance of avoidant death attitudes in realizing healthy emotional outcomes for low to moderate grievers and the need to measure death attitudes when assessing emotional reactions to death. Research and therapy manipulating social rehearsals/interactions or mindfulness training to affect the FAB (or other emotional measures) and grief should also assess death attitudes to determine if they moderate any of the effects.

Although the current study purposely examined fading affect for unpleasant death events and comparably intense pleasant events that occurred roughly at the same time to fairly evaluate the FAB, future research could examine fading affect for pleasant and unpleasant death events. Pleasant death events could occur for grievers who experienced high depression prior to the death of loved ones, such as caretakers of stroke survivors [80] and caretakers of Alzheimer’s patients [81]. Based on the wealth of past FAB research showing that unpleasant emotions fade faster than pleasant emotions described in the current study, we expect that affect will fade faster for unpleasant death events than for pleasant death events. Of course, the issue can and should be resolved empirically.

### Limitations

The current study contained several limitations. For example, some participants did not write down or provide their codenames (though explicitly directed to do so while completing phase 1 online), which inhibited the researchers from matching data from phase 1 to phase 2. One solution to the problem with an online portion is to include a reminder screen for participants to email themselves the codename they created and bring that email (phone or printed) to the in-person portion. Alternatively, researchers could use participants’ experimental computer system (e.g., SONA) IDs to match the two phases of the study, deleting the personally identifying information after phase 1 and phase 2. Another limitation was missing information; participants did not include some critical information, which created large gaps in the data. Future researchers should utilize online tools for collecting data and ensure that participants complete each section before continuing with the procedure to reduce missing data. The retrospective, non-experimental design used in the current study could have been another limitation in that it did not allow for inferences of causation between the predictors (grief, time, and avoidant death attitudes) and fading affect. Future FAB research should use experimental manipulation to create directionality and control for alternative explanations and increase the probability of causality [82].

Limitations also included the circumstances of death in that people perceive a death in a flaming automobile completely differently than a slow, expected death as the result of breast cancer [83]. Future research can address this limitation by examining the effects of death circumstances on FAB. As the current study did not examine a host of maladies (e.g., PTSD), because it was not the focus of the current research, future research could also examine their relation to the FAB.

Finally, researchers initially created the Inventory of Complicated Grief to evaluate clinical populations, so it may have been insensitive to the grief experienced by our participants. This potential limitation is unfounded for two reasons. First, the Inventory of Complicated Grief has been reliably used to measure dysfunctional grief symptoms in non-clinical populations, such as college students, for some time [84,85]. In fact, more recent measures, such as the Inventory of Complicated Grief-Revised [86] and the Persistent Complex Bereavement Inventory [37], have successfully assessed complicated grief symptoms in bereaved college students [37,87,88]. Second, grief interacted with initial event affect and death avoidant attitudes in two different interactions in the current study, which suggests that the measure was sufficiently sensitive to the grief experienced by our participants at various times.

## 6. Conclusions

In summary, we found a robust FAB, such that affect faded more for unpleasant (death) events than for pleasant events, and unpleasant affect faded more for older events than for newer events. Most importantly, grief positively predicted the FAB, which is a positive/healthy emotional outcome. Although this relation was the opposite of the expected one, additional analyses and literature made sense of the effect. Death avoidant attitudes moderated this unexpected effect, such that the Initial Event Affect by Grief interaction was not significant at low levels of death avoidant attitudes, but the effect increased as the individual difference variable increased. The three-way interaction results indicate that moderate levels of grief and strong death avoidant attitudes may be resiliency mechanisms during bereavement, which is in line with the mobilization-minimization hypothesis [18]. Future research should examine if social rehearsals and mindfulness can reduce grief for individuals who report extremely low and high grief. Future research should also evaluate the moderating role of death attitudes for low to moderate grievers, and it should examine the moderating effects of emotional processing speed and emotional attachment. Furthermore, future research should assess fading affect for unpleasant and pleasant death events, and it should control for potential limitations. In conclusion, the results in the current study suggest that individuals can overcome the tragic death of a loved, like the death of a child depicted in the movie Collateral Beauty [89], but they must avoid thoughts of death and grieve, so they can activate the necessary cognitive resources to properly regulate the emotions produced by death.

## Figures and Tables

**Table 1:** Zero order correlations for predictor variables.

Tittle	Variable	1.	2.	3.	4.	5.	6.	7.
1.	DEP	--						
2.	ANX	0.61 **	--					
3.	SRS	0.69 **	0.72 **	--				
4.	GRF	0.19 **	0.28 **	0.21 **	--			
5.	NRC	0.28 **	0.27 **	0.21 **	0.48 **	--		
6.	DA_AV	0.18 **	0.20 **	0.30 **	0.40 **	0.35 **	--	
7.	DA_MA	−0.04	−0.04	0.04	0.02	0.04	−0.14 **	--
8.	Neuro	0.42 **	0.36 **	0.50 **	0.13 *	0.12 *	0.20 **	−0.10 *

Note: DEP = Depression; ANX = Anxiety; SRS = Stress; GRF = Grief; NRC = Negative Religious Coping; DA_AV = Death Anxiety Avoidance; DA_MA = Death Anxiety Mature; Neuro = Neuroticism. * *p* < 0.01. ** *p* < 0.001.

**Table 2:** Fading Affect Bias Regression Coefficients (SE) across Quintiles of Grief Ratings (large positive coefficients represent large FAB).

Quintile (Mean Grief Ratings)	Regression Coefficients (*SE*)
10th (1.53)	0.50 (0.09)
25th (2.00)	0.80 (0.70)
50th (2.30)	0.67 (0.06)
75th (2.53)	0.72 (0.06)
90th (3.26)	0.88 (0.10)

Notes: All *ps* < 0.001.

**Table 3:** Fading Affect Bias Regression Coefficients (SE) across Quintiles (Mean Rating) of Grief and Avoidant Death Attitudes Ratings (large positive coefficients represent large FAB).

Quintile (Mean Grief Ratings)	10th (1.63)	25th (2.00)	50th (2.25)	75th (2.50)	90th (2.88)
10th (1.53)	0.60 (0.12)	0.54 (0.54)	0.50 (0.10)	0.45 (0.12)	0.39 (0.16)
25th (2.00)	0.59 (0.09)	0.58 (0.07)	0.58 (0.07)	0.57 (0.08)	0.56 (0.11)
50th (2.30)	0.59 (0.10)	0.61 (0.07)	0.63 (0.06)	0.65 (0.07)	0.67 (0.10)
75th (2.53)	0.58 (0.12)	0.63 (0.08)	0.67 (0.06)	0.70 (0.06)	0.75 (0.09)
90th (3.26)	0.57 (0.20)	0.70 (0.14)	0.80 (0.12)	0.88 (0.10)	1.02 (0.12)

Notes: All *ps* less than 0.02. Quintile (Mean Avoidant Death Attitudes Ratings). Regression Coefficients (SE).
